# The Role of Extracorporeal Membrane Oxygenation Support in Early Management of Unexplained Life-Threatening Acute Heart Failure Due to Left Atrial Cardiac Paraganglioma

**DOI:** 10.7759/cureus.40853

**Published:** 2023-06-23

**Authors:** Besart Cuko, Olivier Busuttil, Mathieu Pernot, Thomas Modine, Louis Labrousse

**Affiliations:** 1 Department of Cardiology and Cardiovascular Surgery, Hopital Cardiologique de Haut-Leveque, Pessac, FRA

**Keywords:** neuroendocrine tumor, giant pheochromocytoma, acute cardiogenic pulmonary edema, short term mechanical circulatory support, extracorporeal membrane oxygenation support, cardiac paraganglioma

## Abstract

Paragangliomas/pheochromocytomas are uncommon neuroendocrine tumors that arise from chromaffin cells located outside of the adrenal gland. Although cardiac paragangliomas have been observed in all heart chambers, the most prevalent are left-atrial paragangliomas, followed by aortic body tumors. Diagnosis of paragangliomas/pheochromocytomas is mostly achieved with a multimodality approach because of her clinical presentation ranging from incidental findings to refractory acute heart dysfunction. The role of extracorporeal membrane oxygenation support in the early management and diagnosis of unexplained life-threatening cardiogenic shock is rapidly increasing worldwide. However, its clinical utility remains still unclear in intractable heart failure due to primary cardiac paraganglioma. We reported a case of a primary left atrial paraganglioma/pheochromocytoma measuring 34 mm at the maximum diameter in a 58-year-old male patient. The patient presented with acute cardiogenic shock, pulmonary edema, and bilateral stroke. Peripherical mechanical circulatory support, in veno-arterial mode, was rapidly instaured for early management in a life-threatening situation. After normal myocardial function recovery and accurate diagnosis, a surgical approach through aortic and pulmonary artery transection for radical tumor resection and left atrial wall reconstruction was performed. Postprocedural recovery and follow-up at six months were uneventful with excellent neurological recovery.

## Introduction

Paragangliomas/pheochromocytomas are uncommon neuroendocrine tumors that arise from chromaffin cells located outside of the adrenal gland. Although cardiac paragangliomas have been observed in all heart chambers, the most prevalent are left-atrial paragangliomas, followed by aortic body tumors [1]. Often, cardiac paragangliomas/pheochromocytomas required a complex surgical approach and most surgeons have limited experience in treatment. Diagnosis and treatment of paragangliomas are achieved through a multimodality approach and a multidisciplinary team assessment, respectively [2].

## Case presentation

**Figure 1:**
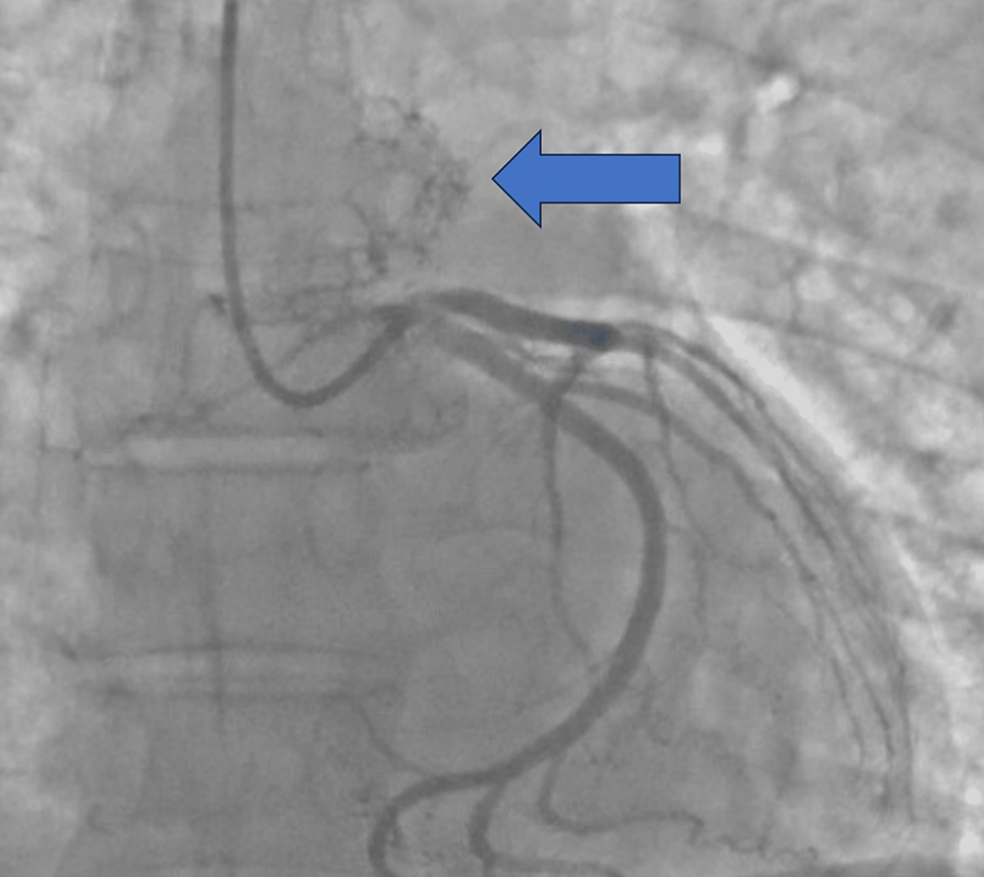
Left atrial mass during coronarography

**Figure 2:**
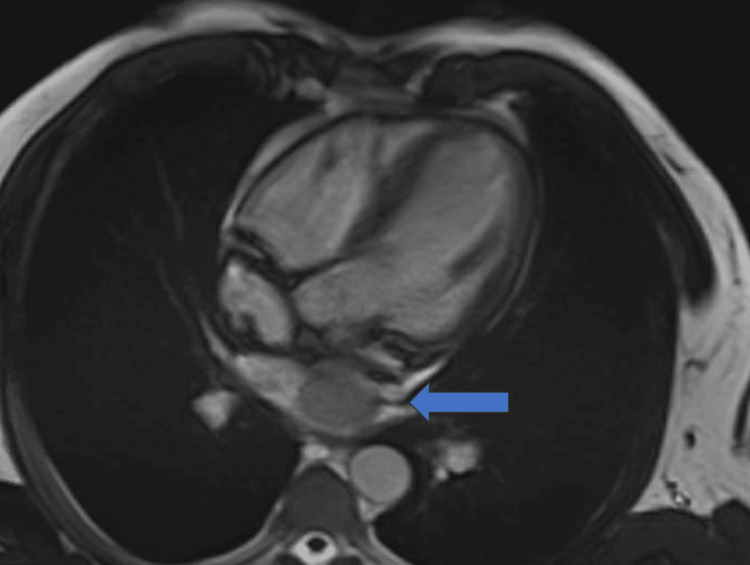
Left atrial mass during cardiac magnetic resonance

**Figure 3:**
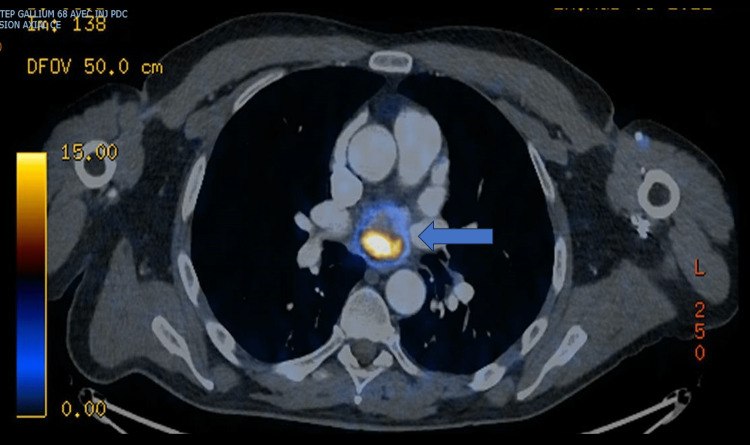
Left atrial mass during positron emission tomography/computed tomography

A 58-year-old male, without known medical history, was initially admitted to our institution for neurological signs, arterial hypertension, tachycardia, and pulmonary edema. Acute coronary syndrome was suspected so diagnostic coronarography was performed with evidence of a neovascularized left atrial mass. During the coronarography, the patient evolved in acute and intractable cardiogenic shock so a peripherical extracorporeal membrane oxygenation support and orotracheal intubation were rapidly instaured. Multimodal diagnosis of the left atrial mass was performed with a diagnosis of primary left atrial paraganglioma/pheochromocytomas measuring 34 mm at the maximum diameter (Figures 1-3).

**Figure 4:**
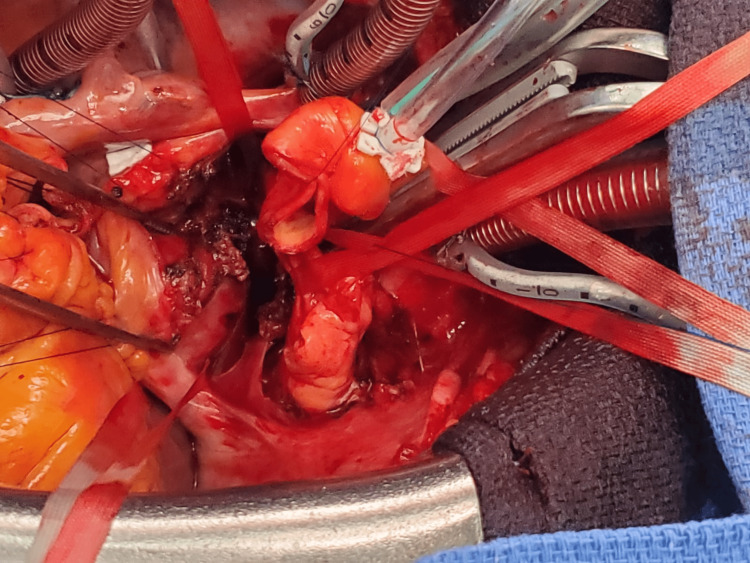
Surgical approach through aortic and pulmonary artery transection

**Figure 5:**
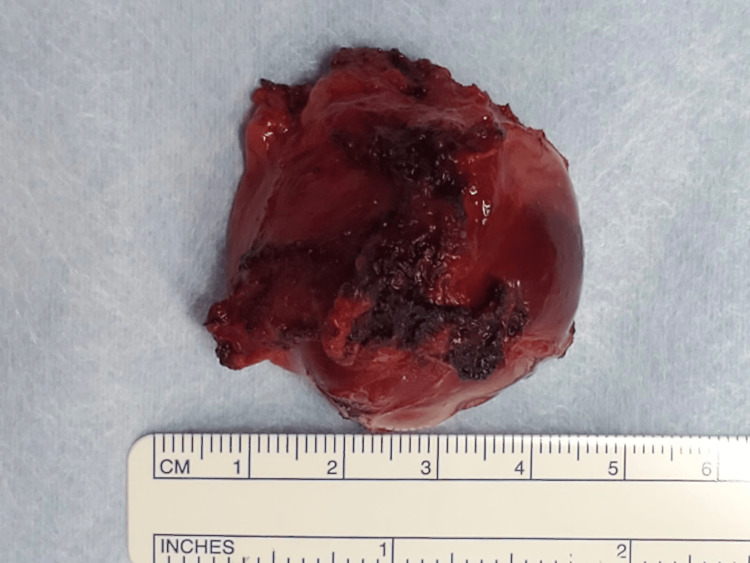
Radical tumor resection

**Figure 6:**
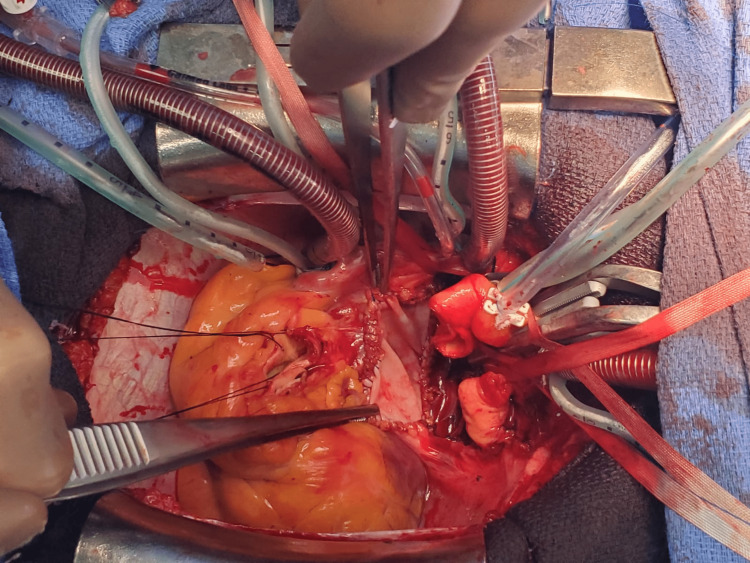
Left atrial wall reconstruction

Weaning of mechanical circulatory support was possible two days after with total myocardial function recovery and hemodynamic stabilization. After the multidisciplinary team assessment, a surgical approach through aortic and pulmonary artery transection (Figure 4) for radical tumor resection (Figure 5) and left atrial wall reconstruction was performed (Figure 6).

**Figure 7:**
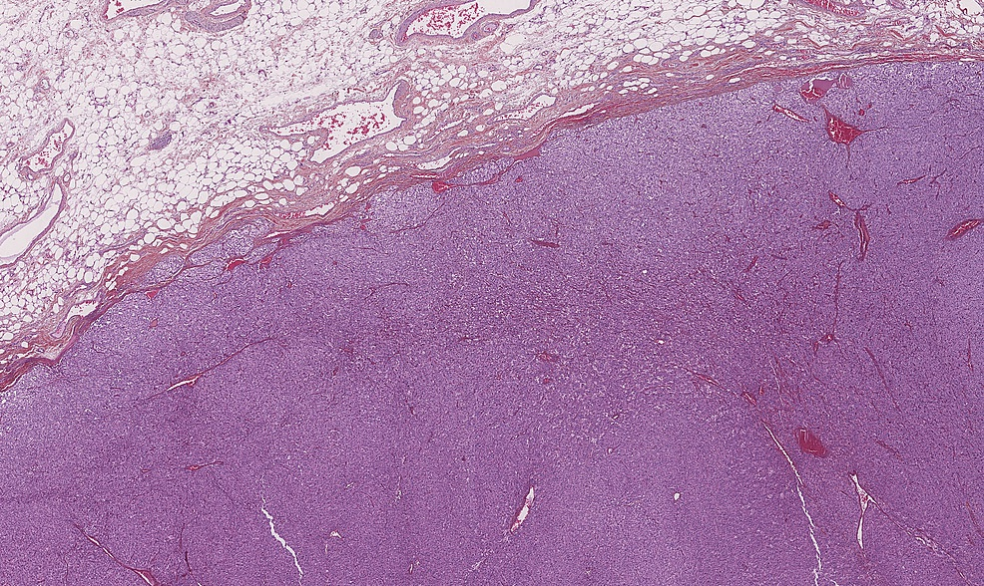
Hematoxylin-eosin staining, magnification ×2.5

**Figure 8:**
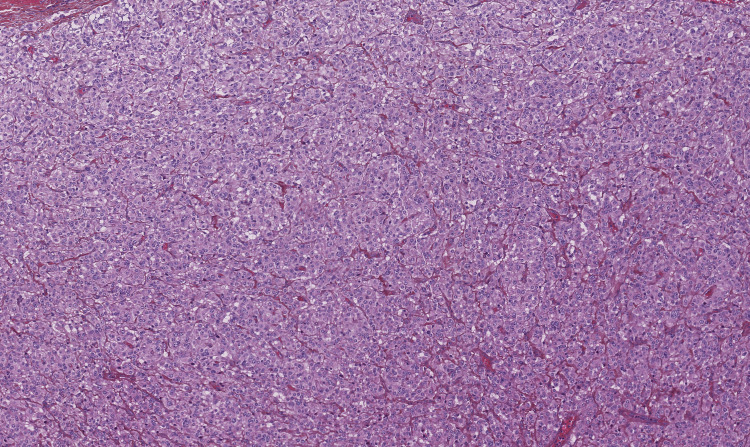
Hematoxylin-eosin staining, magnification ×10

The histologic evaluation was determined to be a cardiac paraganglioma/pheochromocytoma. The specimen was a well-limited, plurinodular proliferation (Figure 7) of small to medium-sized epithelioid cells with granular eosinophilic cytoplasm and nucleoli (Figure 8).

**Figure 9:**
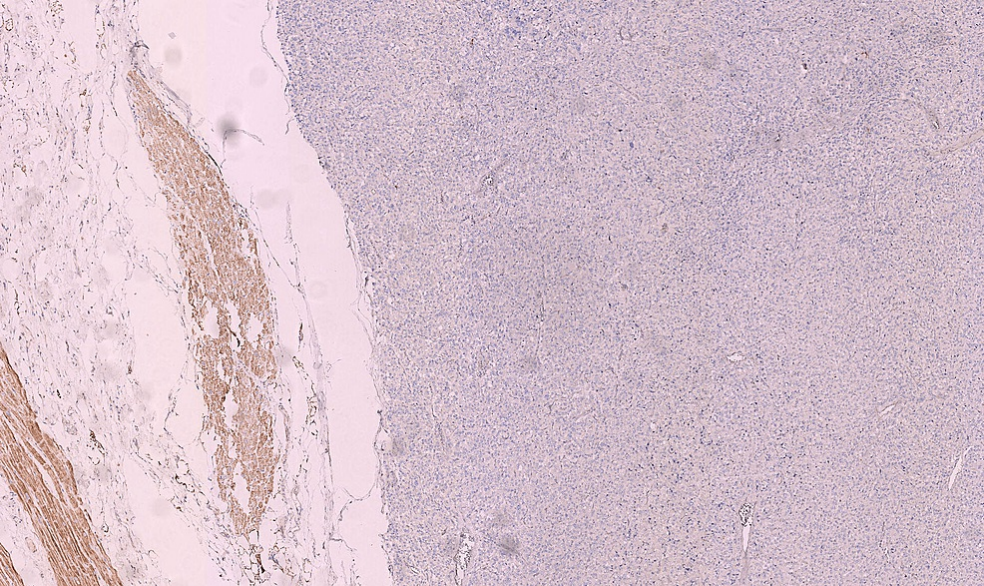
SDHB staining (21A11AE7 clone, made by ABCAM), magnification x5 SDHB: succinate dehydrogenase ABCAM: Cambridge, United Kingdom

The PASS (Pheochromocytoma of the Adrenal gland Scaled Score) was evaluated at 8/20, with a total loss of succinate dehydrogenase (SDHB) expression in immunohistochemistry (Figure 9).

Postprocedural recovery and follow-up at six months were uneventful with excellent neurological recovery. The patient’s informed consent for the procedure and for data collection was obtained.

## Discussion

The clinical presentation of paragangliomas/pheochromocytomas is heavily influenced by the tumor’s metabolic profile, ranging from incidental findings to refractory acute heart dysfunction. In the case of acute cardiogenic shock due to primary cardiac paraganglioma/pheochromocytoma, the clinical utility of extracorporeal membrane oxygenation support in early and life-threatening management still remains unclear even if its role is rapidly increasing worldwide. In a recent systematic review, Matteucci et al. reported the significant role of extracorporeal life support for the successful management of paragangliomas-induced cardiogenic shock, especially in early management for taking time for accurate diagnosis and specific treatment [3]. In the literature, there are also highlights of mechanical circulatory support in endocrine emergencies for the resuscitation of patients with refractory circulatory shock [4,5]. We reported a case of life-threatening cardiogenic shock induced by primary left atrial cardiac paraganglioma/pheochromocytoma treated with peripherical extracorporeal membrane oxygenation support. Weaning of circulatory support was possible two days later, with total myocardial function recovery and hemodynamic stabilization. During and after the circulatory support, we had the possibility of accurate and multimodality diagnosis. A complex surgical approach was performed after a multidisciplinary team assessment with an excellent postprocedural result. We reported this case report to highlight the fundamental importance of extracorporeal membrane oxygenation support in the early management of unexplained life-threatening acute heart failure. We emphasize that gaining time through mechanical circulatory support is crucial for accurate diagnosis and specific treatment, especially in an emergency.

## Conclusions

Extracorporeal membrane oxygenation support is a valuable option in the early management of unexplained life-threatening acute heart failure due to undiagnosed endocrine emergencies, helping restore cardiac function and allowing sufficient time for further, accurate assessment. Multimodality diagnosis and multidisciplinary team assessment of the left atrial mass are fundamental to determining the best surgical approach for total resection of the paraganglioma.

## References

[1] Chan EY, Ali A, Umana JP (2022). Management of primary cardiac paraganglioma. J Thorac Cardiovasc Surg.

[2] Pęczkowska M, Konsek-Komorowska SJ (2022). Cardiac paraganglioma: a challenging diagnostic and treatment dilemma. Kardiol Pol.

[3] Matteucci M, Kowalewski M, Fina D (2020). Extracorporeal life support for phaeochromocytoma-induced cardiogenic shock: a systematic review. Perfusion.

[4] Chao A, Wang CH, You HC (2015). Highlighting Indication of extracorporeal membrane oxygenation in endocrine emergencies. Sci Rep.

[5] Zhou FF, Ding JS, Zhang M, Tian X (2022). Paraganglioma-induced inverted takotsubo-like cardiomyopathy leading to cardiogenic shock successfully treated with extracorporeal membrane oxygenation. Open Med (Wars).

